# A case of pancreaticoduodenectomy and partial hepatic resection as repeat cytoreductive surgery for recurrent pseudomyxoma peritonei

**DOI:** 10.1186/s40792-021-01332-2

**Published:** 2021-12-04

**Authors:** Kenya Yamanaka, Norishige Iizuka, Toshiyuki Kitai

**Affiliations:** 1 Department of Surgery, Hyogo Prefectural Amagasaki General Medical Center, 2-17-77, Higashinaniwa, Amagasaki, Hyogo Japan; 2 Department of Pathology, Kishiwada City Hospital, Kishiwada, Japan; 3 Peritoneal Surface Malignancy Treatment Center, Department of Surgery, Kishiwada Tokushukai Hospital, Kishiwada, Japan

**Keywords:** Pancreaticoduodenectomy, Partial hepatic resection, Repeat cytoreductive surgery, Recurrent pseudomyxoma peritonei

## Abstract

**Background:**

For recurrent pseudomyxoma peritonei (PMP), repeat cytoreductive surgery (CRS) with or without hyperthermic intraperitoneal chemotherapy (HIPEC) can provide survival benefits if patients are carefully selected. We describe a case of pancreaticoduodenectomy and partial liver resection (HPD) as the repeat CRS for a recurrent tumor that infiltrated the pancreatic head around the hepatic hilum. This is the first report of HPD for recurrent PMP.

**Case presentation:**

The patient was a 58-year-old male without comorbidities. In 2001, he was diagnosed with PMP at the time of laparoscopic cholecystectomy. In 2004, CRS, including total peritoneal resection, pyloric gastrectomy, splenectomy, and right hemicolectomy with HIPEC was performed (peritoneal cancer index (PCI) = 28). In 2008, the first repeat CRS with HIPEC was performed (PCI = 14). In 2016, fourth repeat CRS, including partial hepatectomy with HIPEC for recurrence of the round ligament of the liver, was performed. In 2017, a tumor of 5 cm in size was observed from the hepatic hilum to the pancreatic head, which infiltrated the main pancreatic duct. Other tumors 2 cm in size were observed (PCI = 7). We performed the fifth repeat CRS, including HPD. The adhesions of the small intestine from around the liver to the lower abdomen were detached for the reconstruction of pancreatojejunostomy and cholangiojejunostomy. The uncinate approach was applied for the pancreatic head resection because it was difficult to identify the cranial part of the pancreas due to adhesions in the hepatoduodenal ligament and the omental bursa. We approached to the origin of the extrahepatic Glissonean pedicle by resecting a part of the liver around the hepatic hilum using transhepatic hilar approach. A complete cytoreduction was achieved. The postoperative pathological diagnosis was a recurrence of PMP, which is equivalent to peritoneal mucinous carcinomatosis. He was discharged on the 22nd postoperative day without major postoperative complications. The patient survived without recurrence four years after HPD.

**Conclusions:**

Even for recurrence around the hepatic hilum and the pancreatic head, repeat CRS can be safely performed by applying the techniques of hepatobiliary pancreatic surgery.

## Background

Pseudomyxoma peritonei (PMP) is characterized by large amounts of mucinous tumors in the abdominal cavity as a result of appendiceal neoplasms [1–3]. Although curative treatment can be achieved by cytoreductive surgery (CRS) and hyperthermic intraperitoneal chemotherapy (HIPEC), the disease recurrence rate remains high, occurring in 50–80% of cases [4, 5]. For recurrent PMP, with careful patient selection, repeat CRS with or without HIPEC can provide meaningful survival benefits with acceptable mortality and morbidity rates [4–9]. In addition, a significant proportion of patients experience further recurrence, even after the complete removal of recurrent tumors [6, 8]. However, the survival of patients with re-recurrence can improve following a third or subsequent CRS [6, 10].

We describe a case of pancreaticoduodenectomy and partial liver resection (HPD) as repeat CRS for a recurrent tumor that infiltrated the pancreatic head around the hepatic hilum. We believe the present report is significant because it is the first report of HPD for recurrent PMP; moreover, only tumor resection or small bowel resection has often been performed as repeat CRS.

## Case presentation

The patient was a 58-year-old male without comorbidities, whose Eastern Cooperative Oncology Group performance status (ECOG-PS) was grade one. His surgical history was as follows (Table 1): in 2001, he was diagnosed with PMP at the time of laparoscopic cholecystectomy, which was judged to be incurable at the previous hospital. In 2004, he was introduced to us, and CRS, including total peritoneal resection, pyloric gastrectomy, splenectomy, and right hemicolectomy with HIPEC (cisplatin 50 mg) was performed (peritoneal cancer index (PCI) = 28). In 2008, first repeat CRS, including small bowel resection and partial gastrectomy with HIPEC (cisplatin 100 mg) was performed (PCI = 14). In 2011, second repeat CRS with HIPEC (cisplatin 100 mg, mitomycin 20 mg) was performed (PCI = 6). In 2014, a third repeat CRS, including small bowel resection, was performed. In 2016, a fourth repeat CRS, including partial hepatectomy with HIPEC (mitomycin 20 mg) for recurrence of the round ligament of the liver, was performed. At that time, the site around the pancreatic head was ablated via electrocautery, and a postoperative pancreatic fistula was observed.

**Table 1:** Summary of each CRS

CRS	Technique	Area of peritoneal resection	PCI	CC	Period until reoperation (months)
1	Pylorus gastrectomy, splenectomy, right hemicolectomy, omentectomy	Under the left diaphragm, lesser sac, bilateral abdomen, lower abdomen, pelvis	28	1	
2	Small bowel resection, partial gastrectomy		18	0	51
3	Tumor resection, diaphragmatic resection		5	0	31
4	Small bowel resection, tumor resection		3	0	34
5	Tumor resection		2	0	25
6	Partial liver resection, pancreaticoduodenectomy, small bowel resection, tumor resection		7	0	16

*CRS* cytoreductive surgery, *PCI* peritoneal cancer index, *CC* completeness of cytoreduction

**Fig. 1:**
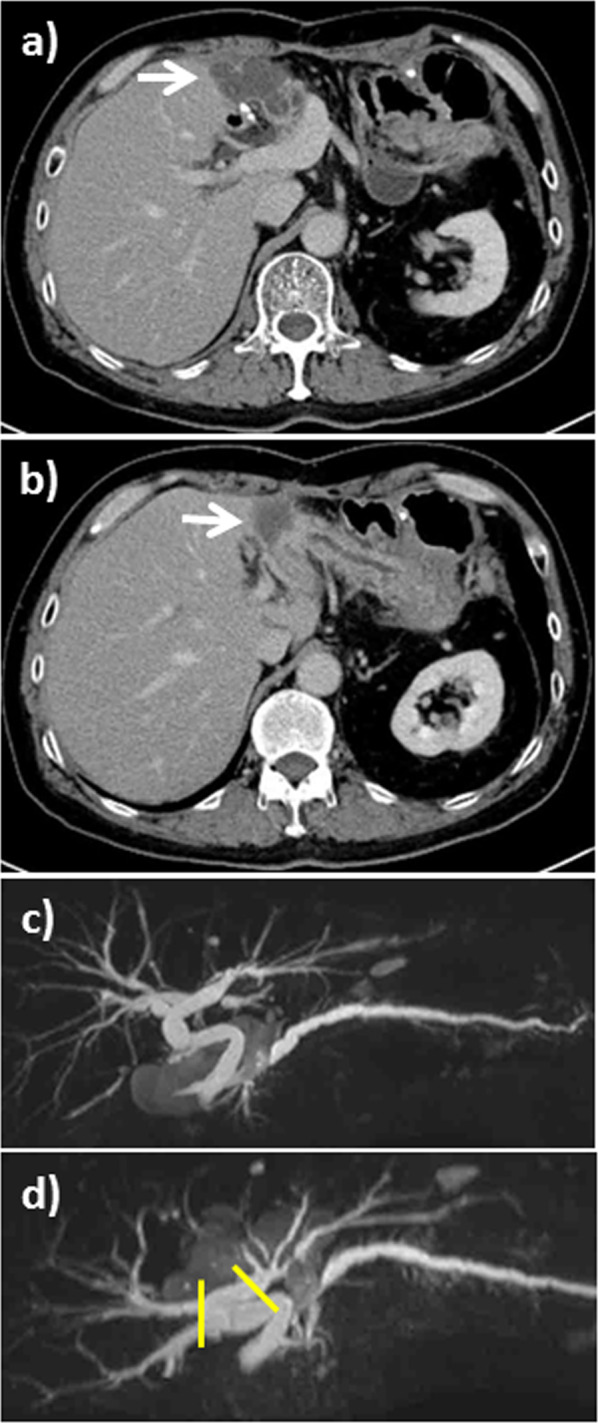
Preoperative enhanced computed tomography and magnetic resonance imaging. **a** The white arrow shows the recurrent tumor in the hepatic hilum. **b** The white arrow shows the recurrent tumor in the hepatic hilum. The main pancreatic duct was dilated. **c** Tumor invasion to the main pancreatic duct and dilatation of the main pancreatic duct on its caudal side. **d** The yellow lines show the resection line of bile ducts

In 2017, enhanced computed tomography (CT) and magnetic resonance imaging (MRI) revealed three recurrent tumors: a tumor 5 cm in size was observed from the hepatic hilum to the pancreatic head, which infiltrated into the main pancreatic duct (Fig. 1a–c). Other tumors 2 cm in size on the lower surface of the liver and on the caudal side of the remnant stomach were observed (PCI = 7). Therefore, we decided to perform the fifth repeat CRS, including HPD.

The abdomen was opened by an inverted T-shaped incision using the incision of the previous operation. We found adhesions of the small intestine from around the liver to the lower abdomen. First, we began sufficient detachment of the adhesions for the reconstruction of pancreatojejunostomy and cholangiojejunostomy. Then, we resected the recurrent tumors on the lower surface of the liver and the caudal side of the remnant stomach.

**Fig. 2:**
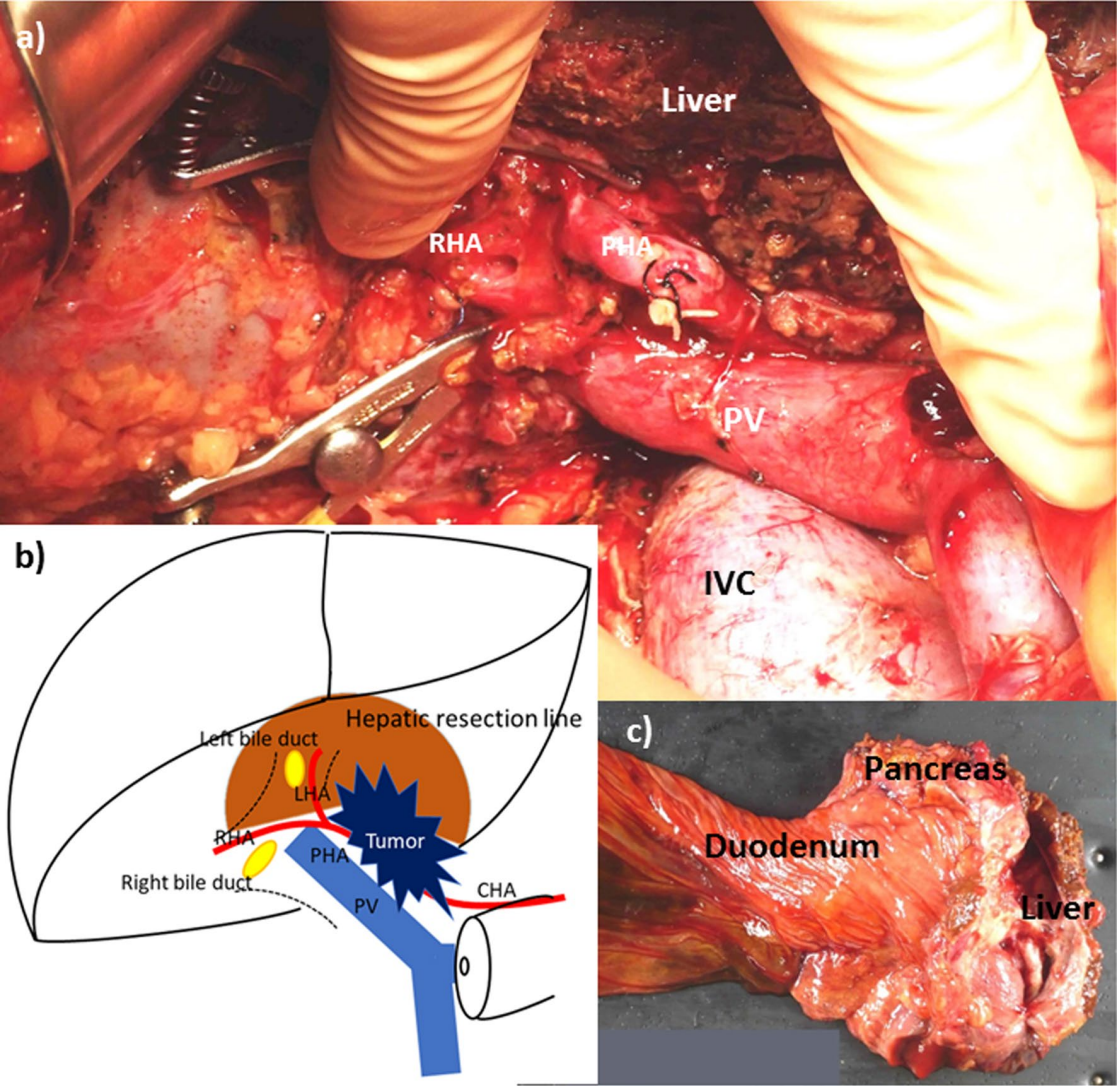
Intraoperative photograph and resected specimen of the pancreatic head and liver. **a** The left and right bile ducts that have been dissected are clamped with bulldog forceps. Lymphatic and connective tissue in the hepatoduodenal ligament remains between the PHA and the RHA. *RHA* right hepatic artery, *PHA* proper hepatic artery, *PV* portal vein, *IVC* inferior vena cava. **b** *LHA* left hepatic artery, RHA: right hepatic artery, *PHA* proper hepatic artery, *CHA* common hepatic artery, *PV* portal vein. **c** The mucus has been removed between the pancreas and the liver

Since it was difficult to approach the pancreatic head directly from the cranial side, we first mobilized the duodenum and pancreatic head from the retroperitoneal adhesions. The jejunum was pulled out to the right and dissected at its origin. We identified the distal side of the superior mesenteric vein (SMV) at the inferior margin of the pancreas and the portal vein (PV) at the back of the hepatoduodenal ligament. The PV and SMV were detached from the posterior surface along with the pancreas. The pancreatic nerve plexus II was dissected along with the pancreas head, then the pancreatic nerve plexus I was dissected on the pancreatic side. We identified the splenic vein and dissected the neck of the pancreas on the PV. After the transection of the pancreas, we identified and encircled the common hepatic arteries. We resected a part of the liver around the hepatic hilum, then exposed and encircled the Glissonean pedicle. The liver resection line is shown in Fig. 2b. The left hepatic artery was identified in the left Glissonean pedicle. Afterward, we identified and encircled the proper hepatic artery and the gastroduodenal artery, and then the gastroduodenal artery was dissected. After subtracting the PV and the hepatic arteries from the encircled Glissonean pedicle, we cut the left and right bile ducts and removed the specimen (Fig. 2a–c). The resection sites of the bile ducts visualized via MRI are shown in Fig. 1d.

Complete cytoreduction was achieved (completeness of cytoreduction (CC) = 0). HIPEC was not performed.

Reconstruction was performed using the modified Child method. A duct-to-mucosal and end-to-side pancreatojejunostomy were used. Cholangiojejunostomy was performed in both segments of the bile duct. The operative time was 697 min, and the blood loss volume was 1453 ml.

**Fig. 3:**
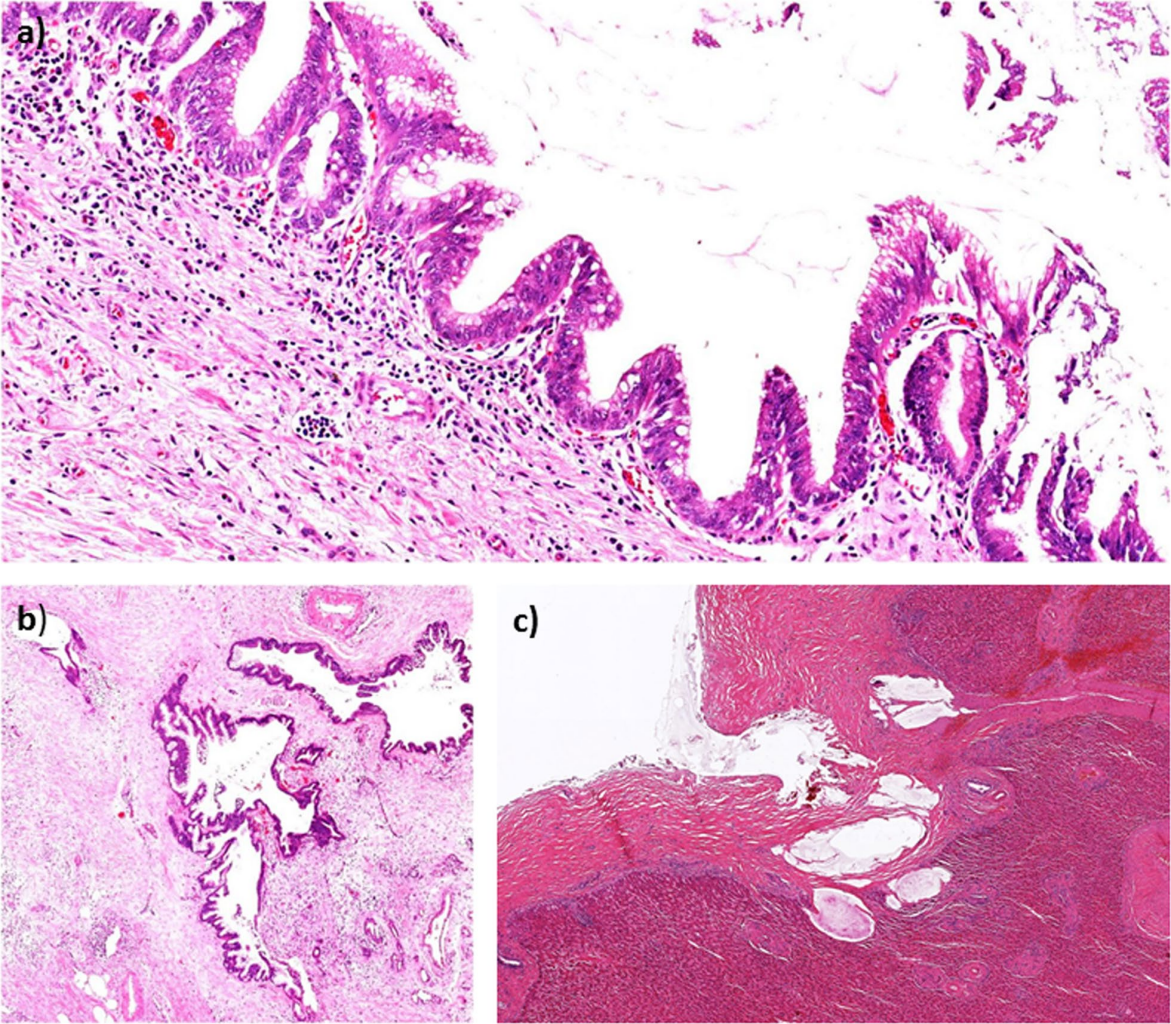
Microscopic pathology. **a** Lesions with nuclear stratification and some degree of atypia, which is equivalent to peritoneal mucinous carcinomatosis, are observed. **b** The mucinous tumor invaded the pancreatic parenchyma destructively. **c** A mucinous lake is observed in the liver parenchyma

The postoperative pathological diagnosis was a recurrence of PMP, which is equivalent to peritoneal mucinous carcinomatosis (PMCA) (Fig. 3a). The mucinous tumor infiltrated into the pancreatic parenchyma destructively, and a mucinous lake was developed in the liver parenchyma (Fig. 3b, c) [11].

A postoperative complication of cholangitis, Clavien–Dindo classification grade 2, was observed. He was discharged on the 22nd postoperative day. Although ECOG-PS deteriorated slightly, he survived without recurrence 4 years after HPD and was followed up at the outpatient clinic.

## Discussion

PMP is a clinic-pathological entity that is characterized by large amounts of jelly-like mucus at some predominated anatomic sites in the abdominal cavity [1–3, 12, 13]. The dissemination process is termed the redistribution phenomenon. Large-volume tumors are often found in the greater omentum, in the pelvis, in the left and right abdominal gutters, and under the surface of the diaphragm [12, 13]. The extent of the disease is assessed via PCI. PCI divides the peritoneal cavity into 13 areas and scores the degree of dissemination in each area from 0 to 3 (0: no tumor, 1: nodules < 0.5 cm, 2: nodules between 0.5–5 cm, and 3: nodules > 5 cm). The total score, ranging from 0 to 39, is obtained by adding all the scores [14]. A complete CRS combined with HIPEC is the recommended treatment option [1, 15]. The Sugarbaker procedure is applied to CRS, in which 1–6 peritonectomy procedures and appropriate gastrointestinal resections are performed to remove all visible tumors with extensive electrocautery [16]. CC is accessed at the end of surgery based on any residual tumor deposits. CC score is classified as either CC0: no residual tumor, CC1: < 2.5 mm, CC2: between 2.5 mm to 2.5 cm, or CC3: > 2.5 cm [15, 17].

Although there are no formal guidelines regarding the indications for repeat CRS with or without HIPEC criteria, patient with favorable tumor biology and the ability to achieve complete macroscopic CRS are usually selected for repeat CRS [5–7, 10, 18, 19]. A PCI score is not a contraindication to attempt repeat CRS as long as signet cells are not present and complete cytoreduction is achieved [5, 8]. Thus, the decision to repeat CRS depends on the following: absence of extraperitoneal metastases, an interval between operations of > 12 months, patient consent, and their performance status (ECOG-PS ≤ 2) [5, 7, 8]. The patient’s ECOG-PS was maintained in this case, and preoperative CT and MRI showed that curative resection could be achieved. The pathological result until the fifth repeat CRS was PMCA without signet ring cells. The interval between operations was 18 months. Therefore, we considered that this case was indicated for repeat CRS including HPD. This management plan has helped the patient survive for more than 4 years without recurrence. More than 20 years have passed since PMP was diagnosed as incurable at the previous hospital.

Repeat CRS is reportedly performed using the Sugarbaker procedure [8, 9]. The small intestine is the dominant site of recurrence and is the most frequently resected organ. Usually, some metastatic nodules on the small intestine and mesentery surface are enucleated or ablated via electrocautery [6]. Urologic procedures, including cystectomy and ureteral resection, have also become more common [4]. However, the Sugarbaker procedure does not mention bile duct resection or pancreaticoduodenectomy. Even if a direct invasion from the peritoneum to the pancreas or the bile duct is not observed on initial CRS, this may appear on repeat CRS because both the extrahepatic bile duct and the pancreas become fragile after peritonectomy. Remarkably, the pancreas is more vulnerable to heat damage than the mesentery of the small intestine [20]. In this case, ablation by electrocautery might have caused pancreatic infiltration after local recurrence of the tumor to require pancreaticoduodenectomy.

Several approaches to pancreaticoduodenectomy have been proposed for pancreatic cancer [21]. The uncinate approach was applied to this case [22]. It was difficult to identify the cranial part of the pancreas due to adhesions in the hepatoduodenal ligament and the omental bursa. Furthermore, we thought that it would be possible to avoid inadvertent cutting into the pancreas by detaching it along the PV. No postoperative pancreatic juice leakage was observed in this case.

We approached the origin of the extrahepatic Glissonean pedicle by resecting a part of the liver around the hepatic hilum. For this approach, we took the transhepatic hilar approach [23]. The Glissonean pedicle was identified and encircled safely at the hepatic hilum, and the bile ducts were identified by subtraction of the PV and hepatic arteries from the pedicle. Since the bile ducts were not directly detached, with some remaining tissue around the bile ducts, cholangiojejunostomy was easily performed. No postoperative bile leakage was observed in this case.

Adhesive detachment is the most important surgical technique in repeat CRS. Aggressive resection of diffuse disseminated tumors in the small intestine and mesentery is associated with more frequent intestinal fistula and postoperative complications [6, 10]. The rate of end ileostomy or colostomy is higher in repeat cytoreductive surgery than in initial surgery [4]. However, few reports mention appropriate methods. Manual and blunt detachment causes unrecognized small bowel damage, which is difficult to repair. Detachment needs to be sharp using electrocautery or scissors (Cooper) under direct vision. Damaged areas should be repaired immediately or marked with thread to be repaired after excision. In this case, it took 3 h or more for adhesion detachment to start after pancreaticoduodenectomy. No postoperative small bowel fistula was observed.

## Conclusions

This is the first report of HPD as repeat CRS for recurrent PMP with a surgical history of 4 repeat CRSs. Even for recurrence on the hepatic hilum and the pancreatic head, repeat CRS can be safely performed by applying the techniques of hepatobiliary pancreatic surgery.

## Data Availability

Not applicable.

## Abbreviations

CC: Completeness of cytoreduction
CRS: Cytoreductive surgery
CT: Computed tomography
ECOG-PS: Eastern Cooperative Oncology Group performance status
HIPEC: Hyperthermic intraperitoneal chemotherapy
HPD: Pancreaticoduodenectomy and partial liver resection
MRI: Magnetic resonance imaging
PCI: Peritoneal cancer index
PMCA: Peritoneal mucinous carcinomatosis
PMP: Pseudomyxoma peritonei
PV: Portal vein
SMV: Superior mesenteric vein
